# Acculturation Timing among Newcomer and more Experienced Immigrant Youth: The Role of Language Use in Ethnic Friendship Homophily

**DOI:** 10.1007/s10964-023-01830-6

**Published:** 2023-08-10

**Authors:** Peter F. Titzmann, Lara Aumann, Richard M. Lee

**Affiliations:** 1 Departement of Psychology, Leibniz University Hannover, Hanover, Germany; 2 Departments of Psychology and Asian American Studies, University of Minnesota, Minnepolis, MN USA

**Keywords:** Acculturation timing, Immigrant adolescents, Host language acquisition, Friendship homophily, Acculturative development

## Abstract

The usage of the new language is a crucial aspect in immigrant youth adaptation. However, despite substantial inter- and intraindividual variability and dynamic changes, language usage has been studied primarily with a focus on static interindividual differences. This study utilized a recently introduced Temporal Model of Acculturative Change to test associations between language acquisition and friendship homophily. More specifically, three concepts were tested: pace (individual rate of change), relative timing (the deviation from peers with similar length of residence), and transition timing (preparedness for the relocation). Data comprised a three-wave-longitudinal sample of 820 ethnic German adolescents from Eastern European States who immigrated to Germany (Mage = 16.1, 57% girls). Results revealed, particularly among recent immigrant adolescents, that transition timing predicted earlier relative acculturation timing in language usage and that early relative timing in language usage predicted levels and change rates in friendship homophily (over and above acculturation pace and the actual level of language usage). Findings highlight the need to better understand the dynamics in acculturation processes of immigrant youth.

## Introduction

Host language acquisition is one of the most pressing challenges for immigrant adolescents as they adapt to a new culture and country (Suárez-Orozco et al., 2018). Host language usage is associated with socioeconomic and psychological adaptation (Guven & Islam, 2015), enhances interethnic communication and conveys a shared identity (Caldas, 2002), and is associated with friendships among immigrant adolescents (Titzmann et al., 2012). Not surprisingly, language acquisition and usage has received substantial attention in immigrant adaptation. However, most studies treat language acquisition and usage as a static interindividual difference score, and ignore the substantial inter- and intraindividual variability and the dynamic changes that take place in language learning. For example, it is important to understand how acquisition of the host language may facilitate or hinder same ethnic friendships, especially if such processes are compared with those in other same-ethnic immigrant peers. Using a recently developed Temporal Model of Acculturative Change (Lee et al., 2020; Titzmann & Lee, 2018, 2022), we investigated the language acquisition and friendship homophily of a unique sample of ethnic Germans from Eastern European States who immigrated to Germany in the late 20^th^ century.

## Temporal Model of Acculturative Change

Scholars studying the adaptation of immigrant youth have recognized the need to integrate developmental concepts on motion and growth into acculturation theory in order to better capture the temporal dynamics of acculturation-related processes (Juang & Syed, 2019; Motti-Stefanidi et al., 2021; Umaña-Taylor et al., 2014). To achieve this aim, Lee and Titzmann introduced a Temporal Model of Acculturative Change that includes various temporal dynamic concepts (Lee et al., 2020; Titzmann & Lee, 2018, 2022). Inspired by the developmental literature on pubertal development (Mendle, 2014; Stumper et al., 2020), the temporal dynamic concepts of acculturative change include Acculturation Timing, Acculturation Tempo, Acculturation Pace, and Acculturation Synchrony (Lee et al., 2020; Titzmann & Lee, 2018, 2022). *Acculturation Timing* is multifaceted and refers to the age at time of migration (chronological timing), the actual start of acculturative changes (transition timing - which may precede or succeed physical migration), and the deviation of acculturative change from relevant others, such as peers from the same cohort and context (relative timing). *Acculturation Tempo* is the duration period of acculturation processes from start to a defined end. *Acculturation Pace* is the speed at which acculturation occurs, which may vary within and between individuals. *Acculturation Synchron*y describes whether adaptation unfolds at the same or different time across different spheres of life. As acculturation is a highly dynamic process, these new temporal concepts allow researchers to better understand the development of immigrant youth adaptation.

While theoretically intuitive and appealing, the Temporal Model of Acculturative Change requires more empirical evidence using longitudinal assessments of acculturation changes among immigrants over long periods of time, ideally, starting before the actual migration. Secondary analyses of existent longitudinal data can provide initial evidence for the predictive power and utility of acculturation timing components. Aumann et al. (2022), for instance, found an accelerated pace in language adoption (i.e., a faster increase in the use of the new language) among immigrant youth predicted higher levels of child disclosures to parents and increased acculturation-related family hassles. These findings provide new insights into the acculturation gap literature and may explain mixed findings in previous studies (Telzer, 2010) by including more dynamic facets of acculturation change. That is, the same acculturation gap between parents and adolescents may have different consequences. If it emerges slowly, parents have time to adapt and intergenerational conflict may be limited or not existent. If such gaps increase more quickly (i.e., through an accelerated adaptation of language use among adolescents), families may experience more intergenerational conflict. This finding on acculturation pace is one piece of the puzzle in studying the dynamics of acculturative change and its consequences. Evidence on other temporal concepts of acculturation remains lacking.

Titzmann and Lee (2022) posit that each temporal concept of acculturative change is likely to be relevant for specific outcomes. For instance, while the study by Aumann and colleagues (2022) found effects of acculturative pace on family interactions, it did not find effects of relative timing – the deviation of immigrants’ adaptation from that of peers with similar length of residence. Aumann et al. (2022) concluded that acculturation pace seems more relevant for explaining family dynamics; whereas, relative acculturation timing (which emphasizes how quickly an immigrant adolescent adopts new behaviors compared to similar peers) may be more pertinent to outcomes that refer to peer processes. The aim of this study, therefore, was to test the effects of acculturative relative timing (i.e., acculturation deviation from immigrant peers with similar length of residence) and pace (i.e., the individual rate of change) on peer relations. We also tested predictors of adolescent relative acculturation timing.

### Relative Acculturation Timing in Language Use and Friendship Homophily

Forming friendships is a central developmental task in adolescence (Havighurst, 1972; Hurrelmann & Quenzel, 2018) and, in modern multicultural societies, this task involves friendship selections across and within ethnicities. Research has found that individuals prefer friends who are similar in characteristics such as age, ethnicity, or gender over dissimilar friends - a phenomenon termed friendship homophily (McPherson et al., 2001). Ethnicity is a particular strong characteristic for friendship selection, because it relates to shared personal and family experiences, such as a common history, language, or cultural community. Due to these commonalities, intra-ethnic friends often provide resources for bonding and emotion regulation. By contrast, inter-ethnic friends primarily provide bridging resources (Serdiouk et al., 2022); bridging resources are associated with knowledge and information about the new society. Both bonding and bridging resources are relevant for young immigrants’ adaptation and explain unique shares of variance in adaptation outcomes (Serdiouk et al., 2022). As moving to a new country is associated with high levels of stress and numerous novel experiences, bonding with intra-ethnic peers can be expected to help with these circumstances and explains high levels of ethnic homophily shortly after arrival in a new country. The shares of intra-ethnic friends (despite significant decreases over time), however, often remain substantial – even after years in the new country (Titzmann & Silbereisen, 2009). Research has also shown substantial inter-individual variation in friendship homophily which is due to differences in attitudes, developmental stages, language use, or contact opportunities (Titzmann, 2014).

For friendship homophily (and for many other outcomes of acculturative change), few empirical studies have investigated adolescents’ timing of acculturation in combination with deviations from peers of similar length of residence. This lack of research is noteworthy, because group norms are fundamental for the psychosocial functioning of adolescents, particularly immigrant youth (Celeste et al., 2016). Peers play a major role in adolescence (McCoy et al., 2019) and comprise a cosmos of mutual influence, norm-setting and socialization (Bukowski et al., 2018). Empirical findings underscore the role of peers in acculturation. One study, for instance, found that deviations from group norms can benefit or harm the psychosocial functioning in immigrant youth (Celeste et al., 2016). Another study revealed more acculturative problems when the acculturation orientations of immigrant adolescents deviated from those of their co-ethnic peers (Titzmann & Jugert, 2015). These findings underscore the role of peers in acculturation processes and highlight that focusing on individual attitudes and behaviors while ignoring such group-processes may limit the understanding of adolescents’ acculturative processes.

The language used in everyday activities is a particular strong factor in the explaining inter-individual differences on friendship homophily. In a multicultural setting, using the host language not only enhances the inter-ethnic communication, but also conveys a shared identity, transports knowledge about the host culture (Caldas & Caron-Caldas, 2002; Gudykunst & Schmidt, 1987), and can ease inter-ethnic spare time activities. Hence, the use of the host language in daily activities is one of the best indicators of sociocultural adaptation to a new cultural setting (Masgoret & Ward, 2006), and past studies have found interindividual differences in host language use explained levels and change rates in ethnic friendship homophily among immigrant youth in Germany and Israel (Titzmann, 2014; Titzmann et al., 2012). Previous research, however, has primarily studied the association between language use and friendship homophily in terms of inter-individual differences.

Importantly, acculturation processes unfold across both the group and the individual levels (Berry, 1997), and, according to the Temporal Model of Acculturative Change (Titzmann & Lee, 2022), the group perspective may add to our understanding of acculturation processes. That is, in addition to the individual level of language use, it may be relevant whether adolescents deviate or not from their peers of similar length of residence and background. Lower levels of use of the new language, for example, can be expected to have different consequences, depending on the group norms. In early stages of immigration, many intra-ethnic peers may not (yet) communicate in the new host language, but after many years some adolescents with lower levels of language use may start to deviate substantially from their peers with consequences for their socio-cultural and psychological adaptation.

This deviation in relative timing of language use may occur in two directions. Adolescents can be early (i.e. they use the new language more often than peers of similar length of residence) or late (i.e. use the new language less often that peers of similar length of residence) in comparison to their immigrant peers. Drawing upon theory and research on pubertal timing, we suggest this differentiation in relative timing of language use leads to two alternative hypotheses, the deviance and the stage termination hypothesis (Petersen & Crockett, 1985). The deviance hypothesis states that being early or late may affect outcomes, because any deviation from group norms has similar consequences. The stage termination theory by contrast assumes that adolescents who are early in language usage compared to peers show differences in adaptation to others. Which of these two hypotheses is supported in relative acculturation timing is not yet known.

### Predictors of Relative Timing in Language Use

Relative timing in the use of a new host language can be expected to have various precursors based on contextual variations and acculturation processes. According to acculturation processes, transition timing seems particularly relevant. Transition timing specifically captures acculturative change when the actual starting point of acculturation does not correspond with the date of immigration (Titzmann & Lee, 2022). Some individuals may prepare for the transition to a new country years before immigration, which may give them a head start in socio-cultural knowledge of the new society, whereas others may delay learning the new language due to contextual circumstances (e.g., living in ethnic enclaves for some time after arrival in a new country). For this reason, indicators for transition timing are associated with levels of preparation to migrate.

Among adolescents, early transition timing can be expected to be associated with adolescents’ participation in the decision to migrate, having the feeling that they were prepared for the transition, and an expressed wish to migrate to the new country. These indicators of early transition timing are expected to be associated with being early in relative acculturation timing. Indicators for late transition timing, such as high shares of intra-ethnic peers in the neighborhood or residing in refugee camps, are probably associated with a delayed start and can be assumed to be particularly relevant for immigrant groups who migrate suddenly, such as refugees.

### Aussiedler Immigrants from the Eastern European States

This study focused on first generation ethnic German diaspora immigrants from the Eastern European States to Germany (Aussiedler). This Aussiedler group provides a unique opportunity to study temporal aspects of acculturation timing, because Aussiedler migrants often identified with Germany before the actual immigration took place. The history of these diaspora migrants reaches back to the regency of Katharina II in Russia (Bade & Oltmer, 2003), when Germans were granted privileges, such as self-administration and cultural-religious freedom, when they settled in Russia. These privileges facilitated German cultural retention and identification with the German homeland.

During World War II, Russian attitude towards ethnic Germans changed substantially, and discrimination, deportations, and a forced adaptation to the Russian culture (Bade & Oltmer, 2003; Dietz, 2000) were the result. In the following decades, ethnic Germans adapted to the Russian mainstream culture, but simultaneously maintained some German cultural practices, especially within the family context (Brenick & Silbereisen, 2012). Nevertheless, adolescent Aussiedler migrants who enter Germany were well-adapted to the Eastern European States and hardly spoke German when they arrived in Germany (Dietz, 2003).

Today, Aussiedler immigrants constitute one of the largest immigrant groups in Germany. As compared to other immigrant groups, they have a somewhat privileged position, as they were granted immediate citizenship, social security, and material support (Dietz, 2000). Hence, in contrast to other refugee groups, these immigrants were more prepared for their immigration due to their history and the process of immigration and acceptance. It is, therefore, an opportune group to study relative acculturation timing – particularly if it comes to early transition timing.

## Hypotheses

The current study aimed to test some of the assumptions of the Temporal Model of Acculturative Change. In a first step, we wanted to test predictors of being early or late in relative timing and included transition timing as a hypothesized variable from the acculturation timing framework as predictor. We hypothesized immigrant adolescents who reported an early transition timing (prepared for the transition, wish to migrate, having participated in the decision to migrate) to be in higher likelihood in the early relative timing of language use (Hypothesis 1). These associations should be independent of other known predictors, such as opportunities for contact as indicated by high shares of intra-ethnic peers in schools (Blau, 1974; Hallinan & Teixeira, 1987), which create intra-ethnic contact and a less established need to talk in the new language.

In the second step, we tested whether relative timing in the use of the new language (early and/or late as compared to peers of similar length of residence and background) as a group-based measure of socio-cultural adaptation predicted friendship homophily in terms of levels (starting values) and change rates (change between assessments) in friendship homophily. Specifically, we hypothesized that being early (stage termination hypothesis) as compared to what is reported by peers of similar length of residence would relate to lower levels of friendship homophily and a less pronounced change rate (Hypothesis 2). That we favored the stage termination over the deviance hypothesis is rooted in the specific group studied: Aussiedler immigrants have German roots and strong support in their adaptation so that we assumed group processes to be particularly relevant if adolescents adjust more quickly as compared to peers of similar length of residence. This hypothesis was based on the assumption that adolescents with early relative timing report levels and change rates in friendship homophily that are usually reported much later in the acculturation process. We also hypothesized (Hypothesis 3) that the association of relative timing in language use is unique and independent from the individual level of language use and ethnic friendship homophily and the individual change rate over the time points (acculturative pace).

Although the associations of the first three hypotheses may be observed at different stages of the acculturative change process, we expected the different timing aspects to be of particular relevance in early phases of the acculturation process, because immigration to a new country can be seen as phase transitions in which even “small fluctuations, or perturbations, have the potential to disproportionately affect the interactions of multiple system elements, leading to the emergence of new forms” (Granic & Patterson, 2006, p. 104). We differentiated between newcomer and experienced adolescents and used 7 years after migration as the cut-off criterion, because parents of adolescent immigrants were found to have a more balanced orientation between their home and their new country, rather than being mainly focused on the country of origin (Birman & Trickett, 2001). In addition, research has found this differentiation to be of relevance empirically in terms of processes and predictions (Titzmann & Silbereisen, 2009, 2012).

## Method

### Sample and Procedure

We used data from a large multidisciplinary and multi-informant longitudinal research project titled “The impact of social and cultural adaptation of juvenile immigrants from the Eastern European States in Israel and Germany on delinquency and deviant behavior.” The original study was funded through the German Israeli Project Cooperation (DIP-4.1) on behalf of the German Federal Ministry of Education and Research (BMBF), Germany. Hence, the study employed secondary data analyses on pre-existing data spanning 3 years, gathered from 2003 to 2006. To investigate our hypotheses, we used adolescent data from Wave 1 to Wave 3.

Participants were recruited from schools. Informed consent was obtained from all adolescent participants in this study and their parents. They were informed about the process and purpose of the research and their right to refuse participation without consequences at any time before, during, and after data collection. Participation in the study was voluntary and participants were told that they were free to withdraw their consent to participate at any time without negative consequences. Inclusion criteria for participation were the following: first-generation immigrant adolescents aged between 10 and 19 years, who were born in the Eastern European States. Adolescents first took part in data collection at school (T1). Later, adolescents were contacted by post in 1-year-intervals, after having consented to their data being used in that way (T2–T3). Those who took part in the first assessment were entered in a prize drawing; all participants in later waves received a 10€ voucher from a statewide electronic store. All procedures performed in this study were in accordance with the ethical standards of the institutional and national research committee and with the 1964 Helsinki declaration and its later amendments or comparable ethical standards. Since we wanted to focus on Aussiedler from the Eastern European States, participants who described themselves as Germans were excluded. Based on the criteria the sample consisted of 1173 Aussiedler adolescents from the Eastern European States. From these, participants who only participated in one measurement point were excluded. As a result, the final sample consisted of 820 adolescents. Based on the criterion of 7 years after migration we differentiated between 391 newcomers and 429 experienced immigrant adolescents. Seventy-eight percent of the adolescents participated at all three measurement occasions. At T1, adolescents’ mean age was 16.1 years (SD = 2.5, range = 10–19 years), and 57% were girls. All Aussiedler adolescents were born in states of the Eastern European States and had a mean length of residence in Germany of 7.4 years (SD = 4.0, range = 1–18 years) at T1.

As is recommended for structural equation modeling, missing values of the remaining participants and of all constructs were handled by the Full Information Maximum Likelihood algorithm (FIML). FIML has been shown to reveal robust estimations if missing data do not exceed 25%, which was not the case in our study (Collins et al., 2001; Newman, 2014). Compared with listwise deletion, the benefit of the chosen procedure is that it does not lead to the common disadvantages, such as losing statistical power or biased parameter estimation (Graham et al., 2003).

### Measures

#### Friendship homophily

We defined friendship homophily as the percentage of intra-ethnic friends out of all friends. Thus, individuals could vary in friendship homophily between 0% and 100%. Participants reported the number of friends that were (a) native Germans and (b) Aussiedler. On average, participants reported a rather large number of friendships (mean between 23.9 and 25.7, median between 17 and 20 depending on the wave of measurement). These numbers indicate that the friendships mentioned included an extended peer network. Friendship homophily was calculated by dividing the number of intra-ethnic friends by the total number of intra- and inter-ethnic friends multiplied by 100.

#### Language use

The mean of two items (“language spoken with friends” and “language used for reading books or journals”) was used to assess the individual level of new language use. Participants rated their language use on a 5-point Likert scale ranging from “never in German” to “always in German” (correlation of items: T1: *r* = 0.52; T3: *r* = 0.49). Two other items originally included in the questionnaire were excluded, namely “language used with parents” and “language of TV shows and radio programs”. These were found to be poor indicators, since the use of the new language with parents and of TV shows depends less on the decision of the adolescents, but rather on the parent’s decision or on the availability of Russian programs (not similar across Germany). The measure of language use was used to create indicators of relative acculturation timing and acculturative pace (see Analyses section).

#### Transition timing

Adolescents reported whether or not they have been prepared both by parents and by others for their migration to Germany (0 = no preparation, 1 = preparation). According to this criterion, 58% of adolescents had been prepared for immigration to Germany. In addition, adolescents reported whether they wished to immigrate to Germany on a 3-point Likert scale (from “didn’t want to come” to “happy to come”). Adolescents also reported who decided to immigrate to Germany – the parents (=0) or the parents together with their offspring (=1). 12% of the adolescents reported they were involved in the decision.

#### Context variables

Adolescents reported their experiences in the acculturation process on a 7-point Likert scale ranging from “only positive” to “only negative” (M = 2.57, SD = 1.2). The share (percentage) of Aussiedler at each school was derived from official school statistics provided by school principals. Adolescents indicated the highest level of maternal and paternal completed education on an internationally comparable scale (International Standard Classification of Education [ISCED]) ranging from 0 (No school level qualification) to 5 (Doctoral degree). The parents were comparably highly educated with 72% having a vocational school degree or higher.

#### Demographic control variables

The adolescents were asked when they migrated to Germany, their age and gender.

### Analyses

Relative timing of language use was established by calculating the average level (mean) trend of language use across each year of residence as well as standard deviations from it. Adolescents, who scored higher than one standard deviation above the mean of all individuals with similar length of residence, were coded as “early” (youth show higher levels of language use than average peer level), whereas adolescents scoring lower than one standard deviation below the mean of all individuals with similar length of residence were coded as “late” (youth show lower levels of language use than average peer level).

Acculturative pace was assessed by using latent True-Change scores (e.g., McArdle & Hamagami, 2001; Steyer et al., 1997). Latent True-Change scores specify “true intraindividual change” scores between two or more occasions of measurement as the values of an endogenous latent variable in the model. In this approach, change over time is modeled directly in the form of latent difference variables, which represent intraindividual changes on latent (hence measurement error-free) level. This also allows to study interindividual differences in intraindividual change (see for example also McArdle & Hamagami, 2001). This measurement model requires factorial invariance (equal loadings and equal intercepts of the indicators) over time, which was established in our case (CFI = 0.99, RMSEA = 0.015, 90% CI: [0.00, 0.02]).

Both relative timing and acculturative pace in language use were used as predictors for friendship homophily in the analyses. To test our predictions concerning friendship homophily, we created a latent linear growth curve model to demonstrate the changes in friendship homophily over the three measurement points (e.g., Ferrer and McArdle, 2003) using the statistical package MPLUS8.9. A two group (newcomer vs. experienced) model was set up with T1 measures as intercepts and a linear slope in both groups. Slope and intercept were allowed to covary, and variances in the manifest measures of friendship homophily were constrained to be equal across the different measurement points in each group. The two group structural equation model showed a good model fit (chi-square = 28.5 [26df], *p* = 0.08; CFI = 0.979, TLI = 0.944, SRMR = 0.029, RMSEA = 0.028) according to common criteria (Hu & Bentler, 1999). In order to test whether the linear model describes our data sufficiently, we tested this model against an unconditional model in which the growth parameters were estimated freely. However, the unconditional model did not significantly improve model fit (chi-square = 82.2, *p* < 0.001; CFI = 0.888; TLI = 0.793; RMSEA = 0.073). Thus, the linear model was the best and most parsimonious in representing our data. Inter-individual differences in intercept and slope of friendship homophily of the growth curve model served as outcomes of the first set of analyses.

Hypotheses were tested in multivariate regression framework. For the prediction of membership in the relative timing groups (Hypothesis 1), we used multinomial regressions in SPSS. For the prediction regarding intercept and slope of friendship homophily (Hypothesis 2) we used linear growth curve models implemented in MPLUS.

## Results

The findings confirmed general processes of acculturative change: Newcomers (length of residence ≤7 years) reported, on average, an intercept of 63.9% intra-ethnic friends out of all friends with a significant longitudinal decrease of 3.5% per year in the study. Experienced adolescents (length of residence >7 years) reported 55.2% of intra-ethnic friends with an insignificant decrease of 1.7% per year. We used multi-group latent-covariate-growth-curve-modelling to examine the similarities or differences of newcomer and experienced adolescents regarding the changes in friendship homophily. To this end, we compared a model where all associations were freely estimated to a model in which paths were constrained to be equal for both groups. The multigroup comparison analysis showed that while the direction of changes were similar across groups, newcomer adolescents reported to have significantly more intra-ethnic friends than experienced adolescents as indicated by a significant chi-square difference test (chi-square = 69.36, *p* < 0.001). Data also showed significant inter-individual variation in intercepts and slopes. Furthermore, among newcomers, length of residence at first assessment was negatively associated with the intercept and positively with the slope in friendship homophily. This association shows a general change in friendship homophily among newcomers, who start with significantly higher levels of friendship homophily and a significantly more pronounced decrease as compared to later stages in the acculturative change process. Among experienced adolescents, no association between length of residence and intercept or slope of friendship homophily was found – meaning that initial general processes of change came more or less to an end and give way to more individualized levels and change rates associated with other variables.

Our first hypothesis predicted immigrant adolescents who reported an early transition timing (i.e., were prepared for the transition, wished to migrate, had participated in the decision to migrate) would have a higher likelihood to be in the early relative timing group of language use (Hypothesis 1). Among newcomers, these expectations were partly met (see Table 1). Specifically, newcomer immigrant adolescents who reported that they were not prepared for the transition had a higher likelihood to be in the late relative timing group and a lower likelihood to be in the early relative timing group. In addition, when newcomer immigrant adolescents reported that they had an explicit wish to immigrate, they had a lower likelihood to be in the late, as compared to the early relative timing group.

**Table 1:** Prediction of membership in the (early or late) relative timing group among newcomer adolescent immigrants

	Late relative Timing				Early relative Timing				Late relative Timing			
	B	Standard Deviation	Sig	Odds ratio	B	Standard Deviation	Sig	Odds ratio	B	Standard Deviation	Sig	Odds ratio
Constant	−5.764	1.472	0.000		4.702	1.508	0.002		−10.467	1.980	0.000	
Age	0.144	0.066	0.028	1.155	−0.395	0.080	0.000	0.674	0.539	0.097	0.000	1.714
Male gender	0.064	0.316	0.838	1.067	0.869	0.309	0.005	2.385	−0.805	0.405	0.047	0.447
Transition Timing												
Wish to immigrate to Germany	−0.169	0.220	0.442	0.844	0.356	0.199	0.074	1.428	−0.525	0.272	0.048	0.591
No preparation by parents for challenges in new country	0.956	0.288	0.001	2.601	−0.450	0.354	0.204	0.638	1.405	0.422	0.001	4.077
Decision by parents	0.275	0.338	0.416	1.316	0.292	0.392	0.457	1.339	−0.017	0.485	0.972	0.983
Context factors												
Percentage of Aussiedler in school at T1	0.028	0.012	0.014	1.029	−0.027	0.013	0.034	0.973	0.055	0.016	0.001	1.057
Positive (0) vs. negative experiences (7)	0.324	0.117	0.006	1.383	−0.061	0.120	0.607	0.940	0.386	0.153	0.012	1.471
Parental education	0.129	0.119	0.280	1.137	−0.249	0.116	0.032	0.780	0.378	0.154	0.014	1.459
Reference group	peer-average				peer-average				early			

In the experienced immigrant group (Table 2), only one association was found between transition timing and relative acculturation timing. Experienced immigrant adolescents who reported an explicit wish to immigrate to Germany also had a lower likelihood to be in the late relative timing group as compared to the early relative timing group – a finding that corresponds to the pattern found among newcomers. In general, we found fewer effects for the experienced immigrant as compared to the newcomer immigrant group, which supports the assumption that relative acculturation timing is particularly relevant in the newcomer phase.

**Table 2:** Prediction of membership in the (early or late) relative timing group among experienced adolescent immigrants

	Late relative Timing				Early relative Timing				Late relative Timing			
	B	Standard Deviation	Sig	Odds ratio	B	Standard Deviation	Sig	Odds ratio	B	Standard Deviation	Sig	Odds ratio
Constant	−5.027	1.435	0.000		0.466	1.634	0.776		−5.492	2.072	0.008	
Age	0.238	0.064	0.000	1.269	−0.119	0.085	0.164	0.888	0.357	0.101	0.000	1.429
Male gender	−0.094	0.310	0.761	0.910	−0.391	0.372	0.293	0.677	0.296	0.460	0.519	1.345
Transition Timing												
Wish to immigrate to Germany	−0.460	0.250	0.066	0.631	0.188	0.145	0.195	1.207	−0.648	0.282	0.022	0.523
No preparation by parents for challenges in new country	−0.128	0.298	0.667	0.880	0.128	0.352	0.717	1.136	−0.256	0.437	0.558	0.774
Decision by parents	0.116	0.661	0.861	1.123	0.085	0.786	0.914	1.089	0.031	0.978	0.975	1.031
Context factors												
Percentage of Aussiedler in school at T1	−0.009	0.012	0.481	0.991	−0.053	0.015	0.000	0.948	0.045	0.018	0.014	1.046
Positive (0) vs. negative experiences (7)	0.366	0.111	0.001	1.442	−0.120	0.147	0.415	0.887	0.486	0.174	0.005	1.625
Parental education	−0.152	0.110	0.167	0.859	0.064	0.125	0.609	1.066	−0.215	0.157	0.171	0.806
Reference group	peer-average				peer-average				early			

It is important to note that these findings controlled for relevant contextual effects, which were more or less similar among newcomer and experienced adolescents. Attending schools with a higher share of Aussiedler increased the likelihood of newcomer and experienced immigrant adolescents to be in the late relative timing group and reduced the likelihood of being in the early relative timing group. In similar vein, newcomer and experienced adolescents who reported negative acculturation experiences reported in higher likelihood to be in the late relative timing group (see Tables 1 and 2). An additional finding was that newcomer adolescent immigrants with more educated parents reported in lower likelihood to be in early relative timing group.

**Table 3:** Prediction of Level (Intercept) and change (slope) in friendship homophily

	Newcomer						Experienced					
	Intercept			Slope (linear)			Intercept			Slope (linear)		
	Estimate	S.E.	*p*	Estimate	S.E.	*p*	Estimate	S.E.	*p*	Estimate	S.E.	*p*
Age	2.550	0.710	0.0001	−0.712	0.489	0.145	1.721	0.570	0.003	0.254	0.389	0.514
Length of residence	−3.593	0.865	0.0001	2.159	0.581	0.0001	1.195	0.781	0.126	0.494	0.541	0.362
Female Gender	0.302	2.458	0.902	0.746	1.938	0.700	1.493	2.681	0.578	0.948	1.865	0.610
Language use T1	−1.482	3.165	0.640	−9.410	2.236	0.0001	−12.467	3.022	0.0001	−1.674	2.412	0.448
Relative Timing late	11.196	6.117	0.067	−6.606	3.894	0.090	−6.883	5.655	0.224	−4.984	4.035	0.217
Relative Timing early	−18.523	6.020	0.002	11.748	3.865	0.002	0.015	5.228	0.998	−2.262	5.228	0.516
Pace T2-T1				−26.788	6.330	0.0001				−42.769	8.784	0.0001

The second hypothesis expected relative acculturation timing in language use to predict interindividual levels and change rates of friendship homophily. Specifically, being early in the use of the new language as compared to what was expected based on the average of adolescents with similar length of residence was hypothesized to relate to lower levels of friendship homophily and a less pronounced change rate. Our findings strongly support this hypothesis among newcomer adolescents. Newcomers who started with higher levels of German language use as compared to the average trend reported significantly lower levels of friendship homophily and a less pronounced decrease – as indicated by the regression weights between early relative timing and intercept and slope of friendship homophily (see Table 3). No association between relative timing and friendship homophily was found among experienced immigrants, which again supported our assumption that relative timing is particularly relevant in initial phases of the acculturation process. These findings were independent of the actual level of language use as well as the individual change in language use over time, which supported Hypothesis 3. The individual level of language use was associated with the slope of friendship homophily among newcomers and the intercept of friendship homophily among more experienced adolescents. The individual change rate over time (which is acculturation pace in terms of the Temporal Model of Acculturative Change) was strongly negatively associated with changes in friendship homophily (slope) in both groups. This finding shows that individual changes in language use (independent of group norms and of being a newcomer or experienced immigrant) are among the strongest predictors for changes in friendship homophily and underscore the role of language in dynamic peer processes.

## Discussion

Language use and friendship homophily are prominent associated indicators of socio-cultural adaptation processes. Hence, these concepts were ideal candidates to test novel concepts derived from the Temporal Model of Acculturative Change (Titzmann & Lee, 2022), which presents different ways to study how acculturation unfolds over time. A core assumption of the model is that not only the level and rate of change must be taken into account, but that these changes must be seen in context of the acculturation process in general, for example when it starts and how co-ethnic peers fare with similar length of residence. This study primarily investigated transition timing and relative timing in acculturation processes. Transition timing is the notion that change may not necessarily start with the actual relocation to another country, but it may precede or succeed the physical migration. Relative acculturation timing refers to deviations from average change processes of peers of similar length of residence (i.e., being early or late). The empirical findings of this study proved the added value of temporal considerations. First, the new concept of transition timing (operationalized by pre-migration preparation and the wish to migrate) predicted relative acculturation timing (i.e., differentiated between adolescents who were early or late in using the new language as compared with adolescents of similar length of residence and heritage). Second, the analyses revealed that early relative timing in the use of the new language was associated with levels and change rates in friendship homophily – particularly among newcomer adolescents. Importantly, these associations were found after individual levels and change rates (acculturation pace) in language use were controlled for. The significance of these well-known control variables shows, however, that language use is associated with friendship homophily in various ways –in terms of associated levels, associated change, and in group deviations.

The findings highlight that a focus on interindividual differences (concurrently and longitudinally) does help in understanding how immigrant adolescents fare in the new society, but that the novel aspects of acculturation timing can add significantly to predictions. More specifically, language use has been identified as construct associated with various outcomes of acculturation (Caldas, 2002; Guven & Islam, 2015; Titzmann et al., 2012), but it is also a variable that is particularly prone for migration preparation. If immigrant adolescents in this study reported the wish to migrate and preparatory actions, they also reported levels and change rates in language use that are typical much later in the acculturation process. In terms of acculturation processes this is a strong indication that adaptation does not start with the physical relocation but can also start much earlier (in our case) or later. In modern societies with new forms of transnationalism (Tedeschi et al., 2022) it is highly likely that length of residence (or chronological timing in terms of the Temporal Model of Acculturative Change) as proxy for acculturation processes will be less relevant and measures of transition timing more prominent to cover the timing of acculturation. The association of relative timing and friendship homophily also adds a group perspective to our understanding of how acculturation unfolds over time. If an adolescent is new in a country and speaks the language exceptionally well (or poorly) the co-ethnic and inter-ethnic context may act with a different understanding as compared to the same level of language use if the adolescents has been in the country for a longer period. Both findings together show that individual acculturation has to be seen more in context and not as a purely individual affair (Bornstein, 2017).

When newcomer (less than 7 years in the new country) and experienced (more than 7 years in the new country) adolescents are compared, we found that the new timing components are particularly relevant for newcomers. These findings indicate that deviations from group norms and considerations of timing are particularly relevant in early stages of acculturation. This finding may support the notion that migration to another country can be seen as phase transitions (Granic & Patterson, 2006) which assumes that life transitions are situations in which “small fluctuations, or perturbations, have the potential to disproportionately affect the interactions of multiple system elements” (p. 104). Among experienced immigrant adolescents, the developmental system may already be stabilized and deviations from the group or differences in transition timing may affect behavior to a lesser extent. An alternative explanation may be that adaptation processes are more uniform initially after arrival in Germany and less so after some time, where proximal contexts and individual choices may increase in relevance. With this reading, the lower effects among experienced adolescents may result from measuring relative timing as the trend across all adolescents in the sample, although the proximal peer context in the specific class or school may be more relevant for assessing relative timing. Unfortunately, we did not have sufficient data to develop a measure of relative timing in language use based on classroom or school peers. Future studies could develop such measures and test whether or not these predict friendship homophily among experienced immigrant adolescents.

Nevertheless, some contextual factors were associated with adolescents’ relative timing in language use – even among experienced adolescent immigrants: The share of Aussiedler immigrants in school and the negative acculturation experiences were associated with later relative timing, a finding that has been published earlier (Blau, 1974; Hallinan & Teixeira, 1987). These associations show that contexts remain important in acculturation processes across stages of adaptation. The exception was that parental education and male gender was only associated with the relative acculturation timing of language use among newcomers. Although the differences between newcomer and experienced adolescents may require more research, it may point out the different roles parents play at different stages of the acculturation process. Among newcomer adolescents, family members are the primary social contacts and hence, parental attitudes (which can be expected to vary with education) may play a more prominent role. Over time, however, adolescents have established more stable peer contacts and parents may play a lesser role (as in all non-immigrating adolescents) (Titzmann & Silbereisen, 2012). In addition, parental education may not be very relevant among experienced parents, because they most likely have changed jobs and may work in professions that are not those they were trained for in the Eastern European States. The effect of male gender being in higher likelihood in the early and less likely in the late relative timing group among newcomers may be explained by the fact that newcomer parents endorse more traditional gender role attitudes and grant more autonomy to boys as compared to girls (Bumpus et al., 2001) so that newcomer males have more opportunities for inter-ethnic contact. Among experienced adolescents, parental gender roles may be less relevant. Taken together, our study supports research that contextual factors are important for acculturation-related processes of friendship formation, but dynamic aspects of acculturation timing can add to this knowledge - particularly in initial phases of the acculturation processes.

Although this study is among the first to test novel assumptions of acculturation timing, it is not without limitations. First, we studied a particular group of immigrants Diaspora Aussiedler immigrants from the Eastern European States in Germany. These immigrants share a German history and (in contrast to refugees) receive citizenship and support upon arrival. For this reason, this group is particularly relevant to study early relative timing. Late relative timing may be more relevant in other groups, such as refugees, who may be prevented from adaptation through extended stays in refugee camps and who face much more juristic obstacles. Second, we tested the association between language use and friendship homophily, because these concepts are theoretically very dynamic and known to be associated - ideal concepts for assessing novel aspects of acculturation timing. Findings may differ substantially with other concepts as described in the Temporal Model of Acculturative Change (Titzmann & Lee, 2022). Third, despite the fact that we have longitudinal data, we cannot draw any conclusions with regard to the direction of effects. Rather, what we observe is co-development (e.g., Buchmann & Steinhoff, 2017; Wrzus & Neyer, 2016) with potentially reciprocal influences between language and friendship formation: On the one hand, speaking the new language may increase the likelihood of interactions with members of other groups who, on the other hand, may improve the language learning. However, the aim of this study was less on the direction of effects, but the predictive utility of novel concepts of acculturative timing. Prospective longitudinal studies that start before the actual migration might be advisable to be informative in terms of causality. Finally, our study has some methodological limitations: It is based on secondary data analysis which has limits in the measurement of some variables and availability of peers in proximal contexts (e.g., the classroom variables). Although secondary data analysis is a good first step, future projects may study acculturation timing prospectively with tailored measures and research designs. Such studies may also profit from testing other timing components. Given our first evidence, this seems a promising line of research.

## Conclusion

The main aim of this study was to demonstrate that acculturation is a dynamic process and that individuals vary in the timing they undergo acculturation-related changes. The Temporal Model of Acculturative Change (Titzmann & Lee, 2018, 2022) can help to structure the role of time in acculturation processes. As in the research on pubertal timing (Mendle, 2014; Stumper et al., 2020), our study was able to show that particularly being early in the language acquisition is associated with friendship homophily and that being early or late is not a matter of chance, but embedded in the process of the transition to the new country (transition timing assessed here through premigration preparedness and an active wish to migrate). Given the novelty of these concepts and the fact that this is one of the first studies to test its assumptions about the dynamics of acculturation, the findings do suggest that time or temporality is an underestimated factor in current acculturation research – a field that is by definition a research field on change (Redfield et al., 1936). The same level of sociocultural adaptation (language use in our case) may have different implications shortly vs. a long time after immigration or in comparison to co-ethnic immigrants with similar length of residence. Processes of change may vary between and within individuals and this variation in dynamic processes requires more attention (see Bornstein, 2017, for example, for the importance of time in acculturation processes). Knowledge on timing is imperative, as it can inform research designs (e.g., spacing between waves in longitudinal research; identification of sensitive life stages for particular developmental processes, the interplay of short-term life changes and long-term plasticity) and will help to better understand mixed results in primarily variable driven research. In this regard, research on the dynamics of acculturation can be informative for other developmental processes that unfold differently within and across individuals.

## References

[CR1] Aumann L, Titzmann PF, Lee RM (2022). Striking a new path to study the adaptation processes of immigrant adolescents: changes in language use and family interactions. Developmental Psychology.

[CR2] Bade, K. J., & Oltmer, J. (2003). *Aussiedler: deutsche Einwanderer aus Osteuropa*. Vandenhoeck & Ruprecht.

[CR3] Berry JW (1997). Immigration, acculturation, and adaptation. Applied Psychology: An International Review.

[CR4] Birman D, Trickett EJ (2001). Cultural transitions in first-generation immigrants: acculturation of Soviet Jewish refugee adolescents and parents. Journal of Cross-Cultural Psychology.

[CR5] Blau PM (1974). Presidential address: parameters of social structure. American Sociological Review.

[CR6] Bornstein MH (2017). The specificity principle in acculturation science. Perspectives on Psychological Science.

[CR7] Brenick A, Silbereisen RK (2012). Leaving (for) Home. European Psychologist.

[CR8] Buchmann M, Steinhoff A (2017). Co-development of student agency components and its impact on educational attainment—theoretical and methodological considerations. Research in human development.

[CR9] Bukowski, W. M., Laursen, B., & Rubin, K. H. (2018). *Handbook of Peer Interactions, Relationships, and Groups (2. Aufl.)*. Guilford Press.

[CR10] Bumpus MF, Crouter AC, McHale SM (2001). Parental autonomy granting during adolescence: exploring gender differences in context. Developmental Psychology.

[CR11] Caldas SJ (2002). A sociolinguistic analysis of the language preferences of adolescent bilinguals: shifting allegiances and developing identities. Applied Linguistics.

[CR12] Caldas, S. J., & Caron-Caldas, S. (2002). A sociolinguistic analysis of the language preferences of adolescent bilinguals: shifting allegiances and developing identities. *Applied Linguistics*, 23(4), 490–514. 10.1093/applin/23.4.490http://search.ebscohost.com/login.aspx?direct=true&db=psyh&AN=2002-08131-003&site=ehost-live.

[CR13] Celeste L, Meeussen L, Verschueren K, Phalet K (2016). Minority acculturation and peer rejection: costs of acculturation misfit with peer-group norms. British Journal of Social Psychology.

[CR14] Collins LM, Schafer JL, Kam CM (2001). A comparison of inclusive and restrictive strategies in modern missing data procedures. Psychological Methods.

[CR15] Dietz B (2000). German and Jewish migration from the former Soviet Union to Germany: background, trends and implications. Journal of Ethnic and Migration Studies.

[CR16] Dietz, B. (2003). Post-Soviet Youth in Germany: group formation, values and attitudes of a new immigrant generation. In T. R. Horowitz, B. Kotik-Friedgut, & S. Hoffman (Eds.), *From Pacesetters to Dropouts: Post-Soviet Youth in Comparative Perspective* (pp. 253–271). University Press of America.

[CR17] Ferrer E, McArdle JJ (2003). Alternative structural models for multivariate longitudinal data analysis. Structural Equation Modeling.

[CR18] Graham, J. W., Cumsille, P. E., & Elek-Fisk, E. (2003). Methods for Handling Missing Data. In Handbook of Psychology, I. B. Weiner (Ed.). 10.1002/0471264385.wei0204.

[CR19] Granic I, Patterson GR (2006). Toward a comprehensive model of antisocial development: a dynamic systems approach. Psychological Review.

[CR20] Gudykunst, W. B., & Schmidt, K. L. (1987). Language and ethnic identity: An overview and prologue. *Journal of Language and Social Psychology*, 6, 157–170. 10.1177/0261927X8763001http://search.ebscohost.com/login.aspx?direct=true&db=psyh&AN=1989-14978-001&site=ehost-live.

[CR21] Guven C, Islam A (2015). Age at migration, language proficiency, and socioeconomic outcomes: evidence from Australia. Demography.

[CR22] Hallinan MT, Teixeira RA (1987). Opportunities and constraints: black-white differences in the formation of interracial friendships. Child Development.

[CR23] Havighurst, R. J. (1972). *Developmental task and education*. David McKay Company Inc.

[CR24] Hu L-t, Bentler PM (1999). Cutoff criteria for fit indexes in covariance structure analysis: conventional criteria versus new alternatives. Structural Equation Modeling.

[CR25] Hurrelmann, K., & Quenzel, G. (2018). *Developmental tasks in adolescence*. Routledge.

[CR26] Juang LP, Syed M (2019). The evolution of acculturation and development models for understanding immigrant children and youth adjustment. Child Development Perspectives.

[CR27] Lee, R. M., Titzmann, P. F., & Jugert, P. (2020). Towards a more dynamic perspective on acculturation research. In P. F. Titzmann & P. Jugert (Eds.), *Youth in Superdiverse Societies. Growing up with globalization, diversity, and acculturation*. (pp. 74–91). Routledge.

[CR28] Masgoret, A.-M., & Ward, C. (2006). Culture learning approach to acculturation. In *The Cambridge handbook of acculturation psychology*. (pp. 58-77). Cambridge University Press. 10.1017/CBO9780511489891.008.

[CR29] McArdle, J. J., & Hamagami, F. (2001). Latent difference score structural models for linear dynamic analyses with incomplete longitudinal data. In L. M. Collins & A. G. Sayer (Eds.), *New methods for the analysis of change* (pp. 139–175). (Reprinted from In File).

[CR30] McCoy, R. T., Pavlick, E. & Linzen, T. (2019). *Right for the Wrong Reasons: Diagnosing Syntactic Heuristics in Natural Language Inference*. 10.18653/v1/p19-1334.

[CR31] McPherson, M., Smith-Lovin, L., & Cook, J. M. (2001). Birds of a feather: homophily in social networks. *Annual Review of Sociology*, 27(1), 415–444. 10.1146/annurev.soc.27.1.415. http://search.ebscohost.com/login.aspx?direct=true&db=psyh&AN=2001-18658-005&site=ehost-live.

[CR32] Mendle J (2014). Beyond pubertal timing: New directions for studying individual differences in development. Current Directions in Psychological Science.

[CR33] Motti-Stefanidi F, Pavlopoulos V, He J (2021). Immigrant youth resilience: Theoretical considerations, empirical developments, and future directions. Journal of Research on Adolescence.

[CR34] Newman DA (2014). Missing data: five practical guidelines. Organizational Research Methods.

[CR35] Petersen AC, Crockett L (1985). Pubertal timing and grade effects on adjustment. Journal of Youth and Adolescence.

[CR36] Redfield R, Linton R, Herskovits MJ (1936). Memorandum for the study of acculturation. American Anthropologist.

[CR37] Serdiouk M, Wilson TM, Gest SD (2022). Cross-ethnic and same-ethnic friendships in elementary classrooms: Unique associations with school adjustment. Journal of Applied Developmental Psychology.

[CR38] Steyer R, Eid M, Schwenkmezger P (1997). Modeling true intraindividual change: True change as a latent variable. Methods of Psychological Research.

[CR39] Stumper A, Graham AA, Abramson LY, Alloy LB (2020). Pubertal synchrony and depressive symptoms: differences by race and sex. Journal of Youth and Adolescence.

[CR40] Suárez-Orozco C, Motti-Stefanidi F, Marks A, Katsiaficas D (2018). An integrative risk and resilience model for understanding the adaptation of immigrant-origin children and youth. American Psychologist.

[CR41] Tedeschi M, Vorobeva E, Jauhiainen JS (2022). Transnationalism: current debates and new perspectives. GeoJournal.

[CR42] Telzer EH (2010). Expanding the acculturation gap-distress model: an integrative review of research. Human Development.

[CR43] Titzmann PF (2014). Immigrant adolescents' adaptation to a new context: ethnic friendship homophily and its predictors. Child Development Perspectives.

[CR44] Titzmann PF, Jugert P (2015). Acculturation in context: the moderating effects of immigrant and native peer orientations on the acculturation experiences of immigrants. Journal of Youth and Adolescence.

[CR45] Titzmann PF, Lee RM (2018). Adaptation of young immigrants: a developmental perspective on acculturation research. European Psychologist.

[CR46] Titzmann PF, Lee RM (2022). New temporal concepts of acculturation in immigrant youth. Child Development Perspectives.

[CR47] Titzmann PF, Silbereisen RK (2009). Friendship homophily among ethnic German immigrants: a longitudinal comparison between recent and more experienced immigrant adolescents. Journal of Family Psychology.

[CR48] Titzmann PF, Silbereisen RK (2012). Acculturation or development? Autonomy expectations among ethnic German immigrant adolescents and their native German age‐mates. Child Development.

[CR49] Titzmann PF, Silbereisen RK, Mesch GS (2012). Change in friendship homophily: a German Israeli comparison of adolescent immigrants. Journal of Cross-Cultural Psychology.

[CR50] Umaña-Taylor AJ, Quintana SM, Lee RM, Cross WE, Rivas-Drake D, Schwartz SJ, Syed M, Yip T, Seaton E, Ethnic Racial Identity in the 21st Century Study Group. (2014). Ethnic and racial identity during adolescence and into young adulthood: an integrated conceptualization. Child Development.

[CR51] Wrzus C, Neyer FJ (2016). Co-Development of personality and friendships across the lifespan. European Psychologist.

